# mRNA vaccines: a new era in vaccine development

**DOI:** 10.32604/or.2024.043987

**Published:** 2024-09-18

**Authors:** SHUBHRA CHANDRA, JENNIFER C. WILSON, DAVID GOOD, MING Q. WEI

**Affiliations:** 1 School of Pharmacy & Medical Sciences, Gold Coast campus, Griffith University, Brisbane, QLD-4222, Australia; 2 Menzies Health Institute Queensland (MHIQ), Gold Coast Campus, Griffith University, Brisbane, QLD-4215, Australia; 3 School of Allied Health, Australian Catholic University, Brisbane, QLD-4014, Australia

**Keywords:** Cancer immunotherapy, Immune checkpoint, Preventive & therapeutic vaccine, Delivery system, mRNA

## Abstract

The advent of RNA therapy, particularly through the development of mRNA cancer vaccines, has ushered in a new era in the field of oncology. This article provides a concise overview of the key principles, recent advancements, and potential implications of mRNA cancer vaccines as a groundbreaking modality in cancer treatment. mRNA cancer vaccines represent a revolutionary approach to combatting cancer by leveraging the body’s innate immune system. These vaccines are designed to deliver specific mRNA sequences encoding cancer-associated antigens, prompting the immune system to recognize and mount a targeted response against malignant cells. This personalized and adaptive nature of mRNA vaccines holds immense potential for addressing the heterogeneity of cancer and tailoring treatments to individual patients. Recent breakthroughs in the development of mRNA vaccines, exemplified by the success of COVID-19 vaccines, have accelerated their application in oncology. The mRNA platform’s versatility allows for the rapid adaptation of vaccine candidates to various cancer types, presenting an agile and promising avenue for therapeutic intervention. Clinical trials of mRNA cancer vaccines have demonstrated encouraging results in terms of safety, immunogenicity, and efficacy. Pioneering candidates, such as BioNTech’s BNT111 and Moderna’s mRNA-4157, have exhibited promising outcomes in targeting melanoma and solid tumors, respectively. These successes underscore the potential of mRNA vaccines to elicit robust and durable anti-cancer immune responses. While the field holds great promise, challenges such as manufacturing complexities and cost considerations need to be addressed for widespread adoption. The development of scalable and cost-effective manufacturing processes, along with ongoing clinical research, will be pivotal in realizing the full potential of mRNA cancer vaccines. Overall, mRNA cancer vaccines represent a cutting-edge therapeutic approach that holds the promise of transforming cancer treatment. As research progresses, addressing challenges and refining manufacturing processes will be crucial in advancing these vaccines from clinical trials to mainstream oncology practice, offering new hope for patients in the fight against cancer.

## Introduction

RNA therapeutics, an innovative domain in the field of medicine, has the potential to transform the treatment of various diseases fundamentally. This groundbreaking approach centers on harnessing ribonucleic acid (RNA) molecules, the genetic couriers accountable for essential cellular functions in our bodies [1]. Despite the existence of RNA therapies dating back to the introduction of the antisense oligonucleotide (ASO) Vitravene in 1998, achieving clinical and commercial success has been a complex and non-linear journey [2,3].

The emergence of the COVID-19 pandemic played a crucial role in advancing the adoption and advancement of an additional RNA technology known as messenger RNA (mRNA) [4,5]. The remarkably rapid progress in developing COVID-19 vaccines demonstrated the safety, effectiveness, and scalability of mRNA. This success also sparked a revitalized enthusiasm for RNA technologies, leading to an elevenfold surge in merger and acquisition (M&A) activity between 2020 and 2021 [6].

RNA therapies have the advantage of being chemically synthesized, unlike biological products that may necessitate intricate setups and bioreactors. This implies that once a promising RNA candidate is identified, it can undergo rapid production and testing. The versatility of RNA technology adds to its appeal. There is currently a considerable level of interest in RNA therapeutics, with entities of various sizes actively involved in the development and testing of candidates [7].

**Figure 1:**
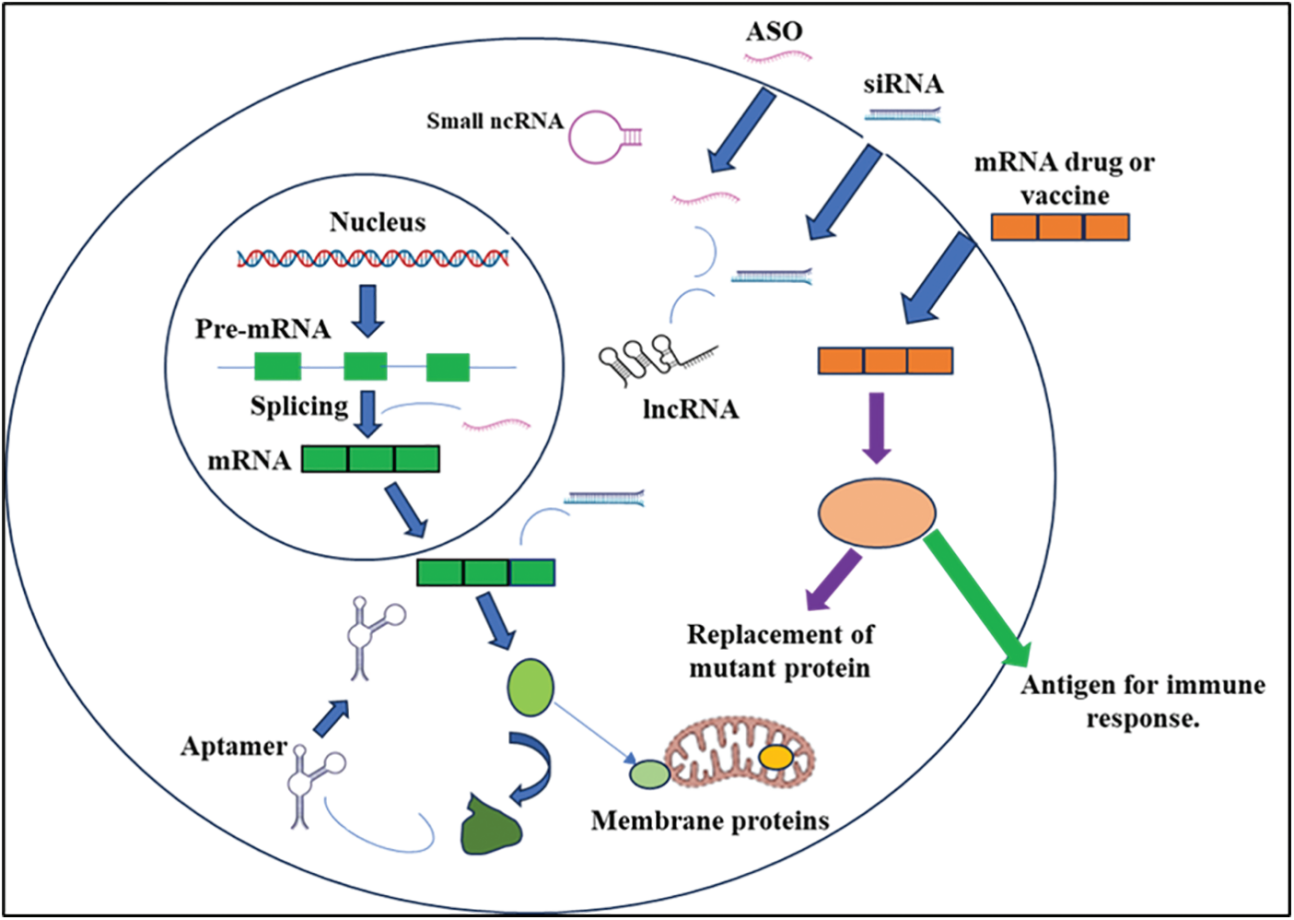
RNA-based medications have the capacity to intervene at various stages of protein-coding and noncoding gene expression. Splicing manipulation is achievable through antisense oligonucleotides (ASOs), while mature messenger RNAs [9] can be targeted using ASOs or small interfering RNAs (siRNAs). Moreover, noncoding RNAs (ncRNAs), encompassing both small and long forms (lncRNAs), can be suppressed utilizing ASOs or siRNAs. Aptamer binding enables the modulation of protein function. Additionally, exogenous mRNAs can be employed to introduce specific proteins into cells, either to restore deficient enzymes or to serve as antigens, triggering a targeted immune response.

The prevalent RNA types under development include siRNAs, ASOs, and mRNAs, constituting 80% of the RNA therapeutics pipeline [8] (Fig. 1). This observation is unsurprising, as these three RNA platforms are the most advanced, having approved products and proven commercial success. Notably, only 19% of the pipeline is classified as orphan medicines, indicating a departure from the majority of currently marketed RNA-based drugs. The future of RNA therapeutics appears promising for larger patient populations.

Certainly, the most extensively explored therapy area is cardiometabolic disorders, constituting almost one-third of the entire pipeline. Among RNA classes, small-interfering RNA (siRNA) takes the lead. An exemplary instance is Rivfloza, created by Novo Nordisk, which received FDA approval in October 2023 for treating primary hyperoxaluria in adults and is currently in the process of being introduced to the market [10]. Another noteworthy siRNA therapy is Eli Lilly’s candidate lepodisiran, which recently received positive news following the results of its initial human trial [11]. The findings, disclosed at the AHA conference, demonstrated an impressive reduction of up to 94% in lipoprotein(a) (LP(a)) levels with a single dose of the drug, lasting nearly a year. LP(a) is a well-established risk factor for cardiovascular disease.

Following siRNAs, the subsequent significant category comprises ASOs, making up 28% of the cardiometabolic RNA pipeline. Although ASOs may be the earliest type of RNA to have been introduced to the market, there is certainly no decline in innovation. Ligand-conjugated antisense (LICA) oligonucleotides merge an ASO with a ligand, resulting in a product with heightened specificity and more precise delivery to the targeted area of the body [12]. Eplontersen, currently in the pre-registration phase with the FDA after acceptance for a New Drug Application, is an example of a LICA oligonucleotide. Developed by Ionis for a subtype of amyloidosis known as ATTRv-PN, it shows promising progress [13].

It’s important to highlight that close to a quarter of the candidates in the cardiometabolic domain focus on diseases related to the liver. The liver stands out as an attractive organ for RNA therapies because delivering medication to it is more straightforward compared to other organs, thanks to its function in the body and its elevated metabolic activity [14].

Oncology ranks as the second-largest field for RNA advancements. In this domain, the predominant RNA technology is mRNA, constituting 32% of the pipeline, closely followed by ASOs at 29%. Notably within oncology, some of the most promising developments involve therapeutic mRNA vaccines [15]. These vaccines entail the identification of either patient-specific or tumor-related antigens, followed by the manufacturing and administration processes. Many of these products are currently undergoing trials alongside checkpoint inhibitors, such as Moderna’s mRNA-4157/V940. The combination with pembrolizumab demonstrated a significant 44% improvement in recurrence-free survival for melanoma patients [16].

### The path ahead

The increasing presence of RNA therapeutics addressing both rare and common diseases currently undergoing clinical trials reflects our enhanced comprehension of fundamental biology and, notably, our ability to convert these insights into human medications [17]. However, in an increasingly challenging pharmaceutical market, compelling science alone may not be adequate. To effectively progress from clinical development to a commercial product, those driving RNA innovation must internalize certain key lessons.

Innovations in primary care: RNA assets that are either marketed or in the advanced stages of development frequently focus on disease areas with substantial patient populations, a departure from the prevailing trend of mostly approved orphan drugs [18]. In the realm of primary care, RNA faces a challenging landscape, not only contending with a frequently generic and cost-effective standard of care (SoC) but also confronting a mindset among payers and prescribers that often favors a “good enough” approach that doesn’t necessarily reward innovation [19]. Hence, innovators must distinctly articulate the distinctions between RNA therapies and the standard of care, quantifying the value they bring to both patients and health systems.

Forward momentum in innovation: Health systems find it challenging to match the increasing number of specialty and orphan drug launches. Concurrently, the level of innovation grows more intricate, with multiple advanced therapeutics occasionally vying for prominence in the same domain—some even presenting curative methodologies. For RNA innovators, ensuring the robust readiness of health systems is crucial as part of their targeted launch strategy. This is essential to secure a robust uptake within a potentially limited window of commercial opportunity [20].

Undoubtedly, RNA therapies present numerous advantages compared to small molecule or biological drugs, and there is a strong momentum in innovation due to the rapid evolution of the science [21]. Coupled with a string of success stories in the commercial realm, the overall outlook for the entire field is optimistic. Vaccines play a key role in preventing the spread of diseases and saving lives. The use of conventional vaccines, including live attenuated and inactivated viruses and subunit immunizations, has led to a decrease in the prevalence of infectious diseases such as smallpox, polio, and measles. However, there are still barriers to expanding vaccine coverage against virulent diseases, especially those that can evade the adaptive immune response. Additionally, traditional vaccination methods might not be appropriate for treating non-contagious diseases like cancer. As a result, it is necessary to develop more efficient and adaptable vaccination platforms to address these challenges [22].

Over a decade, significant scientific advancements and research breakthroughs have positioned mRNA as a favourable treatment option for vaccine production and protein substitution, offering a new frontier in medical innovation [23]. Compared to other types of vaccines, including subunit, dead, live attenuated viral vaccines, and DNA-based vaccines, mRNA offers various benefits. Firstly, it has a reduced chance of infection or insertion mutagenesis as it is non-infectious and non-integrating. mRNA is also naturally degraded by physiological mechanisms, and its duration of activity can be controlled through changes in delivery strategies [24]. The intrinsic immune response elicited by mRNA can also be reduced to enhance its overall safety profile. Secondly, changes in mRNA can lead to a more viable and translatable form, which can be delivered effectively *in vivo* using a desired carrier vehicle for faster absorption and expression in the cytoplasm. mRNA is the smallest genetic vector and can be given frequently, with the added benefit of eliminating anti-vector immunity. The high yields produced through *in vitro* transcription processes also make mRNA vaccines a promising option for quick, low-cost, and scalable production [25].

mRNA vaccines offer a novel approach to cancer therapy by using genetic material to instruct cells to produce antigens specific to cancer cells. This triggers an immune response, targeting and destroying cancer cells [26]. Encouraging results from early-stage clinical trials have validated the capability of mRNA vaccines for different forms of cancer, such as melanoma, breast, and prostate cancer [27]. The FDA has authorized two mRNA vaccines for prophylactic use. One of these vaccines is designed to target the hepatitis B virus and could also assist in preventing liver cancer resulting from this virus, while the other targets cancers associated with the human papillomavirus (HPV), including cervical cancer [28]. These vaccines have demonstrated remarkable efficacy in avoiding infection and lowering the likelihood of cancer. However, more research is needed to determine their efficacy and safety in larger populations and advanced stages of cancer. Additionally, vaccines based on mRNA technology are still in the initial phases of growth and are not yet extensively available as a cancer treatment option [29].

The most important factor in designing an mRNA vaccine is to make sure that the desired tumor antigen is expressed at a high level. There are many options for tumor antigens in carcinomas, so there are many potential candidates for inclusion in vaccines. Currently, there have been multiple clinical trials of mRNA vaccines for cancer treatment, including successful phase 1/2 trials for non-small cell lung and prostate cancer by CureVac AG. mRNA vaccines offer a safer and more cost-effective alternative to other immunotherapy options such as surgery, chemotherapy, and radiotherapy. Additionally, mRNA vaccine production is scalable and can be easily produced [30].

### mRNA-based vaccine strategy

The field of mRNA vaccine research has been growing rapidly in recent decades, especially for the treatment and prevention of cancer [27]. Scientists have made progress in establishing and enhancing tumor-specific T-cell responses, although significant challenges remain. Despite many efforts, the progress for therapeutic cancer vaccines is limited, with only one vaccine approved by the FDA to treat prostate cancer [31]. Sipuleucel-T (known as Provenge^®^ or APC8015) represents an innovative cancer vaccine derived from autologous dendritic cells (DC) that are infused with a specially engineered fusion protein consisting of prostatic acid phosphatase (PAP) and granulocyte-macrophage colony-stimulating factor (GM-CSF). Early trials in Phase I and Phase II have demonstrated the vaccine’s safety and its ability to elicit immune responses directed at the fusion-protein target antigen, also referred to as PA2024. Recent Phase III investigations have further validated Sipuleucel-T’s effectiveness in extending median survival among patients with CRPC (castration-resistant prostate cancer), albeit with minimal impact on clinical disease progression or surrogate markers such as serum PSA kinetics. Consequently, the United States Food and Drug Administration has granted approval for Sipuleucel-T for the management of asymptomatic or minimally symptomatic CRPC [32]. One approach that has been studied is nucleic acid therapy, which has advantages over traditional vaccine methods. Initial success with mRNA vaccines was reported in 1990 using *in vitro* transcribed [33] mRNA, and a study by Jirikowski et al. demonstrated that mRNA in the rat hypothalamus produced a functional response [34]. However, mRNA vaccines have faced issues such as instability, poor *in vivo* administration, and strong innate immune response. To address these problems, researchers have been incorporating nucleotides and protein-based components to promote proper protein folding and full functionality, as well as immune-specific adjuvants, which have shown improved antigen specificity [35,36]. In general, mRNA vaccines provide several advantages compared to vaccines made from killed viruses, live-attenuated viruses, or subunit DNA. Firstly, they are non-integrating and non-infectious, therefore least possibility of infection or insertional mutagenesis. The mRNA’s duration of activity can also be monitored and manipulated through alterations in delivery methods [37–40]. Secondly, mRNA stability and translational ability can be improved by modifying nucleosides. Thirdly, the *in vivo* delivery efficiency of mRNA vaccines could be improved by incorporating the mRNA into a carrier molecule for quick uptake and expression in the cytoplasm. Additionally, mRNA vaccines do not have a genetic vector, so there is no risk of an anti-vector immune response, making them a safe choice. Finally, mRNA vaccines can be produced efficiently and inexpensively due to the elevated production of mRNA *in vitro* through transcription [41].

### The structure and method of delivering mRNA vaccines

**Figure 2:**
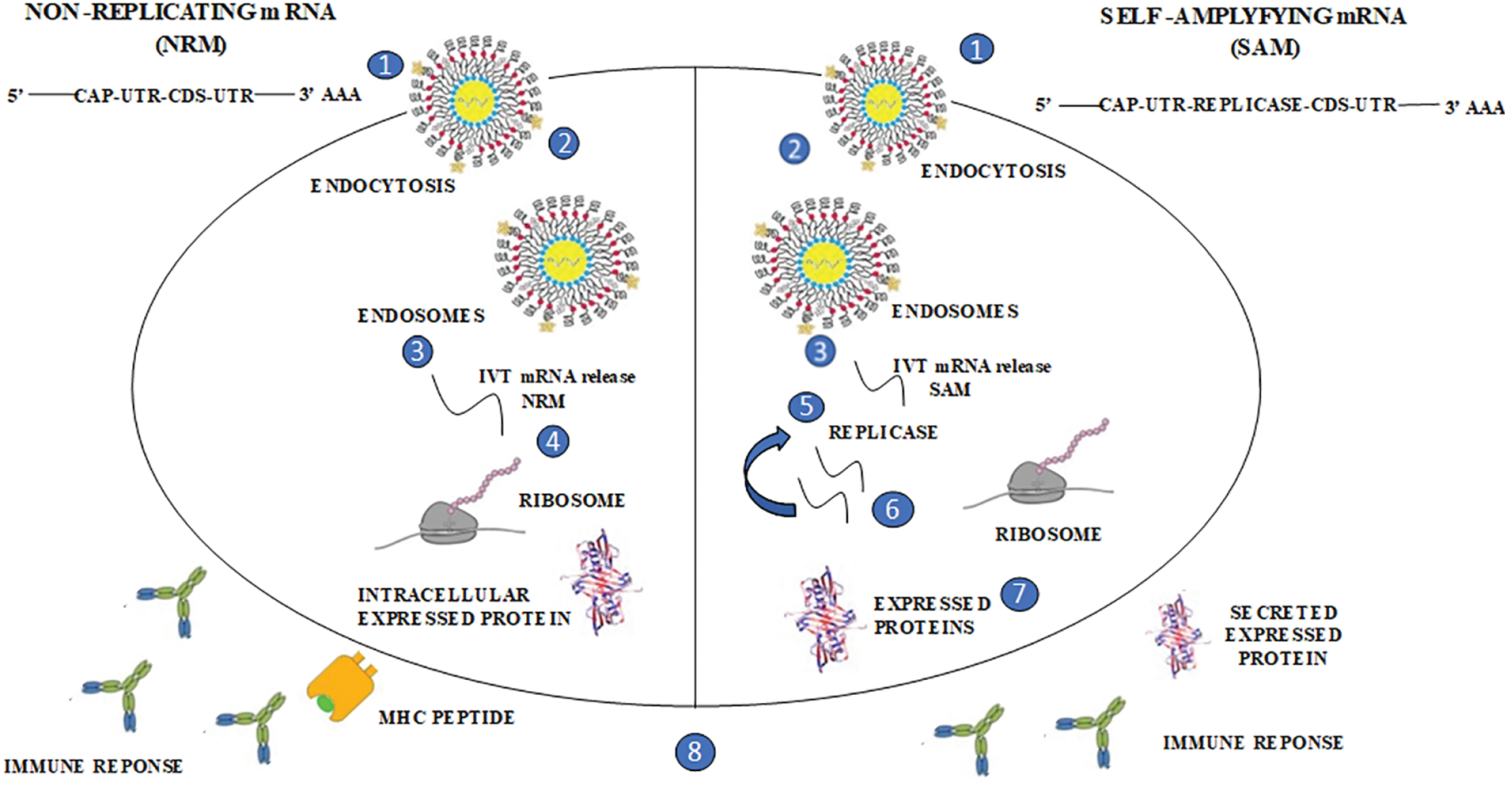
The distinctive forms of mRNA structures, non-replicating mRNA (NRM) and self-amplifying mRNA (SAM); The structure of SAM consists of an untranslated region (UTR) and coding sequence (CDS), like the structure depicted in the provided image. However, SAM is identified by the existence of a replica, which enables prompt intracellular amplification of mRNA. In the image, this progression is represented as follows: (1, 2) A lipid nanoparticle is employed to prevent degradation and facilitate cellular uptake. Through this delivery system, SAM utilizes the (3) endocytic pathway, (4) allowing the mRNA to be circulated into the cytosol. SAM’s unique structure leads it into the cytosol, where ribosomes translate the construct, guiding the expressed protein for post-translational modification. (5) SAM’s replicase machinery aids in the translation process by ribosomes, which is crucial for the self-amplification of RNA. The expressed proteins undergo (6) post-translational modification, and these proteins are subsequently sorted to either the (7) trans/intracellular membrane or (8) secreted. Both the innate and adaptive immune responses recognize these proteins.

The idea behind using mRNA technology for creating vaccines is to use a virus to present the desired transcript, which encodes the target immunogen, in the host cell’s cytoplasm. This leads to the expression of the translated proteins, which can be either secreted or located within the cell. Currently, there are two distinct forms of mRNA: non-replicating mRNA (NRM) and self-amplifying mRNA (SAM) structures which are being explored. The common features of both non-replicating mRNA (NRM) and self-amplifying mRNA (SAM) structures include the 5′ and 3′ untranslated cap structure regions (UTR), an open reading frame [42], and a 3′ poly A tail [43]. However, SAM is different from NRM in that it has genetic duplication machinery obtained from positive-strand mRNA viruses, such as Sindbis and Semliki-Forest viruses [44,45]. The ORF coding for the viral structure proteins is replaced with the desired transcript, which includes viral RNA-dependent RNA polymerase. This substitution allows for cytoplasmic expansion of the replicon structure (Fig. 2).

### The process of producing vaccines using mRNA technology

To begin the production of mRNA vaccines, plasmid DNA (pDNA) is first generated comprising of a DNA-dependent RNA polymerase promoter such as T7 [46], with mRNA construct sequence. The pDNA is then linearized and transcribed by DNA-dependent RNA polymerase and later broken down by a DNAse step. The add-on of the 5′ cap and 3′ poly(A) tail can be attained using enzymes like guanylyl transferase and poly-A polymerase, resulting in either Cap 0 (N7MeGpppN) or Cap 1 formation (N7MeGpppN2′–OMe) [47,48].

Secondly, the ends of mRNA, 5′ and 3′ UTRs, are crucial for achieving maximum gene expression. The effectiveness of gene expression is determined by the specific nucleotide sequence and modifications in the 5′ and 3′ UTRs, which contain regulatory factors.

The third factor to consider is the 5′ 7-methylguanosine (m7G) cap for the molecule of mRNA. This cap is important for efficient translation and prevents degradation by 5′–3′ exonucleases. The composition of the cap is important for generating protein along with immune response. Inadequate capping of 5′ triphosphate and cap 0 structures promotes RIG-1, which inhibits gene expression of the target mRNA. Additionally, mRNA molecules with 2-O’-unmethylated caps are prone to degradation by IFN-induced proteins and tetratricopeptide repeats (IFIT1), which hinder the beginning of translation [49,50]. It is crucial for producers of mRNA vaccines to consider factors such as the selection of enzymes and reaction conditions to ensure optimal cap formation. Moreover, the poly-A tail and its characteristics play a critical role in the translation and protection of the mRNA molecule [51,52].

Lastly, the process of translation can be influenced by codon optimization and modified nucleotides. For example, increasing the content of guanine and cytosine (GC) has been shown to significantly enhance translation as has been demonstrated in DNA vaccines [53]. Regarding its applicability to delivery systems, mRNA’s intrinsic activation may affect gene expression. Modified nucleosides can alter antigen expression or the adaptive immune response. Nucleosides that have undergone modifications like pseudouridine or N-1-methylpseudouridine can be utilized, for instance, to prevent intracellular signaling that activates protein kinase R (PKR) [43,54,55]. Optimizing coding sequences and removing unwanted inflammatory impurities can lead to minimal inflammatory reactions during protein synthesis and notable adaptive immune responses throughout the body [38,40].

The mRNA is purified using HPLC under high pressure [56], and then the final product is defined and distributed based on sterility, identity, purity, and efficacy. These procedures comply with Good Manufacturing Practices and enable the production of new vaccines within a limited timeframe.

To sum up, the impact of the factors mentioned earlier on immunogenicity has been demonstrated through comparative studies, leading to valuable insights for vaccine design.

### Regulation of immune response

To start with the body’s innate immune reaction, exogenous patterns called pathogen-associated molecular patterns (PAMPs) are recognized by pattern recognition receptors (PRRs). The mRNA cancer vaccine aims at antigen-presenting cells identifiable by PRRs, and the exogenous. *In vitro* transcription [33] mRNA is generally immunostimulatory because it’s recognized by diverse cytosolic PRRs, including those found on the cell surface and endosome.

Within the endosome, IVT mRNA is detected by toll-like receptors (TLR7/8), which subsequently trigger the myeloid differentiation factor (MyD) 88 pathway, initiating the type-1 interferon pathways and cytokine production [57].

**Figure 3:**
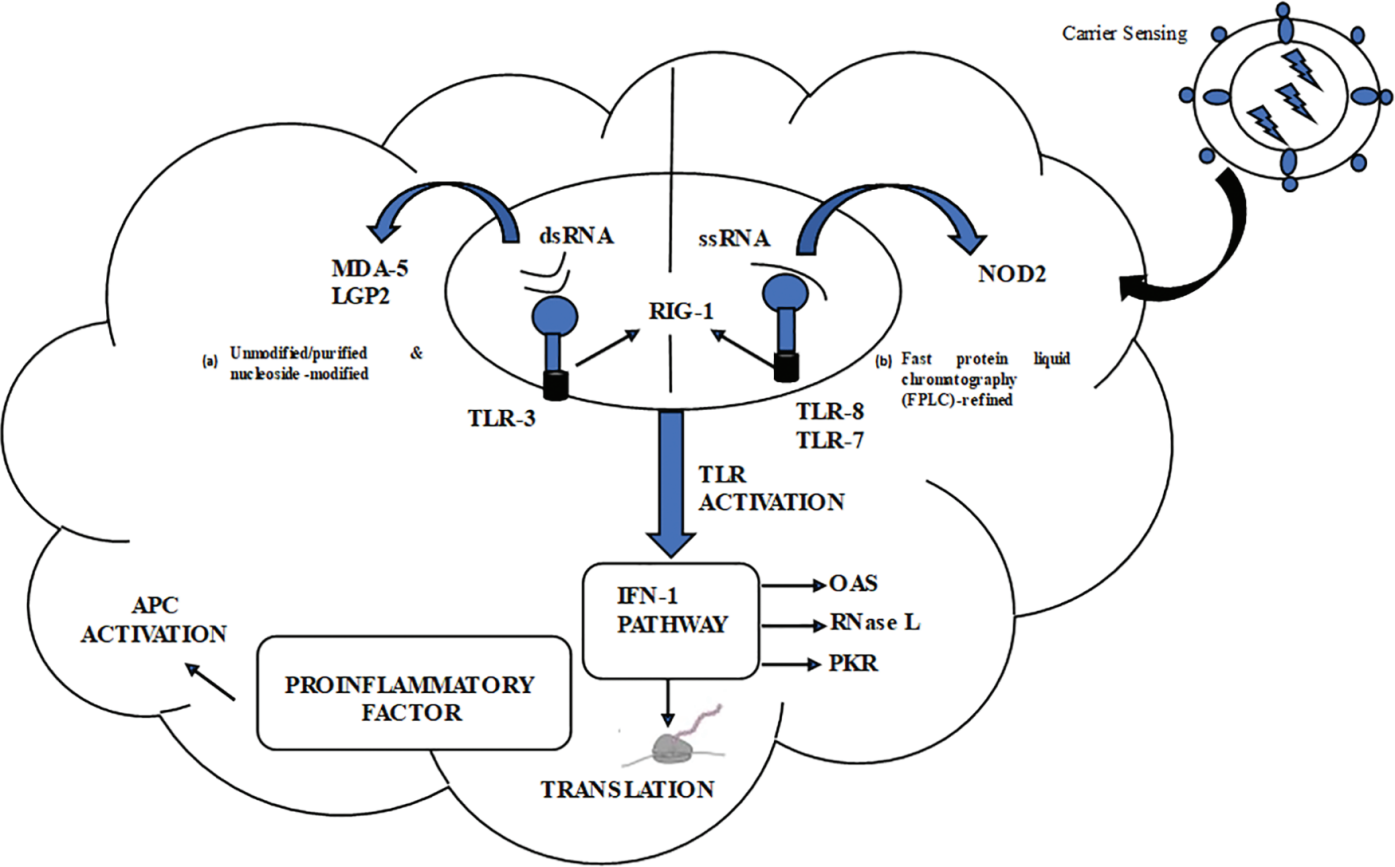
Designing mRNA Vaccines to Equilibrate Innate and Adaptive Immune Responses. The figure illustrates the innate immune response associated with two categories of mRNA vaccines. Blue cogs represent RNA sensors; MDA-5, and LGP2, while blue blub shaped with black tails represent DC maturation factors. In the top right area, a lipid nanoparticle carrier is illustrated. On the left side, there is a list of key RNA sensors responsible for distinguishing double-stranded and unaltered single-stranded RNAs. The figure showcases two formats for mRNA vaccines: (a) Unmodified/purified and nucleoside-modified (b) fast protein liquid chromatography (FPLC)-refined.

In contrast, PRRs found in the cytosol, including PKR and similar receptors such as retinoic acid-inducible gene-1 (RIG-1), recognize and respond to different types of RNA, such as single-stranded, double-stranded, and those that interfere with translation. The distinguishing characteristic of PRRs is their ability to distinguish between different types of RNA (Fig. 3).

A line between the two formats indicates a decrease in antigen expression levels. The abbreviations used in the figure are as follows:

Ag: Antigen.

PKR: Interferon-induced, double-stranded RNA-activated protein kinase.

MDA-5: Interferon-induced helicase C domain-containing protein 1 (also known as IFIH1) IFN: Interferon.

OAS: 2′–5′-oligoadenylate synthetase.

TLR: Toll-like receptor.

Triggering of numerous PRRs and formation of type 1 IFN might have both positive and negative effects on anti-cancer immunotherapy. Occasionally, the stimulation of type 1 IFN pathways can enhance the stimulation and development of antigen-presenting cells, improve antigen presentation, and lead to strong adaptive immune responses, which may be beneficial for vaccination purposes. In other words, although the innate immune system has the capability to identify RNAs, it might also lower antigen expression, leading to a delayed immune reaction. In the process of *in vitro* transcription [33], double-stranded RNA (dsRNA) could be generated, triggering innate immunity via various pathways such as PKR, OAS, TLR-3, and MDA-5 (a type of RIG-1 receptor). Activation of PKR leads to phosphorylation of eukaryotic initiation factor which can result in the inhibition of mRNA translation [58], while dsRNA binding to OAS triggers RNAaseL [59], resulting in the breaking down of foreign RNA molecules. Combining dsRNA with MDA-5 and TLR-3 can ultimately initiate Type I IFN, leading to the activation of numerous other genes that limit mRNA translation [60]. Apart from dsRNA impurities, an mRNA structure that has not been well-designed might also trigger PRRs such as MDA-5 and PKR, resulting in a reduction of the antigen expression. The impact of type 1 IFN stimulation is not limited to antigen expression alone and has an opposing effect on the production of CD8+ T cells. Prior research has extensively explored the dual impact of type 1 interferons (IFNs) on cellular immunity targeting CD8+ T cells [61]. To gain a deeper understanding of the interaction between IFNAR and TR signalling pathways, and how mRNA cancer vaccines might influence this interaction, it is vital to consider the timing and dynamics of their activation. The stimulatory or inhibitory effects of type 1 IFNs on CD8+ T cell generation are thought to be contingent upon these variables. Several studies have suggested that type 1 IFNs could potentially augment the CD8+ T cell response to systemic mRNA immunization [62,63]. One conjecture is that antigen expression and presentation coincide with TCR signals occurring either before or concurrently with IFNAR signalling in splenic DCs, often facilitated by Lipoplex cation. In dermal mRNA vaccination (via intradermal or subcutaneous routes), antigen presentation occurs proximal to the injection site, while antigen presentation takes place in secondary lymphoid organs, where IFNAR signalling precedes TCR signals [64]. Therefore, optimizing mRNA sequences for purity, devising an efficient delivery system, and selecting appropriate administration routes are crucial steps to effectively activate innate immunity while minimizing potential overstimulation that could impede antigenic protein expression and immune response.

### Progress in administering mRNA vaccines

The effectiveness of a potential vaccine relies on efficient *in vivo* uptake of mRNA, which must traverse the lipid membrane and reach the cytoplasm for protein decoding. The effectiveness of mRNA uptake *in vivo* is crucial for the efficacy of a potential vaccine. The method of mRNA uptake varies depending on the cell type [9]. Physicochemical characteristics such as charge, solubility, and optical density can impact mRNA translation and cellular delivery. Two primary methods for mRNA uptake into cells exist. The first involves *ex vivo* insertion of mRNA onto DCs and reintroduction of the genetically modified cells [9], while the second method entails the inoculation of mRNA directly, with or without the assistance of a carrier. *Ex vivo* DC packaging ensures the specificity of the mRNA to the cellular target. It improves transfection efficiency and can be valuable for cell therapies but is costly. In contrast, the administration of mRNA through direct injection is fast and economical but can generate an unpredictable immune response [62]. Table 1 shows the different delivery approaches that have been investigated.

**Table 1:** Various approaches for *in vivo* use of mRNA vaccine

Delivery approach type	Species	Delivery path	Objective
Commercial transfection mixture	Mice	IN	Ovarian cancer [65]
Cationic nanoemulsion	Mice, rabbit, ferret, and rhesus macaque	IM	Influenza virus [66], RSV [67], human immunodeficiency Virus-1 [67,68] herpesviridae [67], *Streptococcus* spp. [69], and rabies virus [70]
Cationic polymer liposome	Mice	IV	Melanoma [71,72], pancreatic cancer [73]
Cationic lipid nanoparticle	Mice	ID, IV & SC	Human immunodeficiency Virus-1 [74] and ovarian [9].
Cationic lipid, cholesterol nanoparticle	Mice	IV, SC. & IS	Influenza virus [62,75], melanoma [62,76], moloney murine leukemia virus, ovarian cancer, human papilloma virus, and colon cancer [62]
Cationic lipid, cholesterol, PEG nanoparticle	Mice, cotton rat and rhesus macaque	ID, IM & SC	Zika virus [43,77,78], influenza virus [79–82], respiratory syncytial virus [83], herpesviridae, rabies virus [70] and melanoma [84]
Dendrimer nanoparticle	Mice	IM	Influenza virus, Ebola virus, *Toxoplasma gondii* [85] and Zika virus [86]
Protamine	Mice, ferrets, pigs and human	ID	Influenza virus [87,88], melanoma [89], non-small-cell lung cancer [90], prostate cancer [91,92], rabies virus [93], Ovarian [88,91,94] and Lewis lung cancer [94]
Protamine liposome	Mouse	IV	Lung cancer [95]
Polysaccharide unit	Mice and rabbit	SC	Influenza virus [96]
Cholesterol/DPPC/PS at a molar ratio of 1/4/5	Mouse	IV	Viral infection
DOTMA/DOPE at a 1:1 molar ratio	Mouse, human	IV	Tumor
Lipid coating: EDOPC/DOPE/DSPE-PEG: 49/49/2, molar ratio Polymer-RNA center: PbAE	Mouse	IV	Tumor
Ionizable lipid/DSPC/cholesterol/PEG-lipid in a molar ratio of 50/10/38.5/1.5	Mouse Ferret non-human primate human	IV	Viral infection, tumor
DOTAP/cholesterol at a molar ratio of 2:3	Mouse	IV	Tumor

Note: IN: Intranasal, IM: Intramuscular, IV: Intravenous, ID: Intradermal, SC: Subcutaneous.

### Loading of DCs ex vivo

DCs are a type of immune cell that perform as effective antigen-presenting cells. They trigger the adaptive immune response by taking in and displaying antigens through proteolysis and exhibiting them to CD8+ T cells by MHC (I), and CD4+ T cells by MHCII. Additionally, DCs can present entire antigens to B cells to stimulate antibody production [97]. DCs are highly receptive to mRNA transfection, making them an ideal target for *in vivo* and *ex vivo* mRNA transfection.

Although DCs can take up non-modified mRNA through the endocytic pathways [98–100], *ex vivo* transfection efficiency can be improved by using electroporation. This technique involves the application of a high-voltage pulse to create membrane pores that allow mRNA molecules to enter the cytoplasm. Electroporation is preferred because it can achieve efficient transfection without the requirement for carrier molecules. *Ex vivo*-loaded DC vaccines are created by combining DCs with mRNA, which enables the vaccine to target and stimulate an immune response. Many *ex vivo*-loaded DC vaccines have been shown to generate a cell-mediated immune response, formulating these as a promising option for cancer treatment [101].

### In vivo administration of self-amplifying mRNA via Interdermal or internodal vaccination

Antigen-presenting cells have efficiently recognized naked mRNA that was administered *in vivo* through intradermal or intranodal injection [98,102–105]. Recent studies have shown that intranodal injection of pure naked mRNA encoding tumor-associated neoantigens led to strong T-cell responses with enhanced progression-free survival [106].

### In vivo methodology for delivering to visceral organs

Different methods have been utilized to enhance the efficiency of mRNA absorption inside living organisms by penetrating the cell membrane. One strategy entails employing gold particles to transport mRNA via a gene gun (a microprojectile technique), which facilitates the distribution of mRNA across tissues [107]. This method has been proven to be effective for RNA delivery and vaccination in mice models, although no human data is available. *In vivo*, electroporation has also been demonstrated to enhance the absorption of therapeutic RNA [108–110]. However, this visceral delivery method has limitations, such as inducing cell death and limited contact with target cells. Recently, nanoparticles made from lipids or polymers have shown promise as effective delivery vehicles [24].

### Methodology for delivering mRNA vaccines.

#### Protamine

Studies have shown that the cationic peptide protamine can guard mRNA against degradation by serum RNases [83]. Nevertheless, when mRNA is paired with protamine [91,111], it can result in reduced levels of protein and the ability of the cancer vaccine to be effective. One potential reason for this decrease in efficacy is the short linker between protamine and mRNA. This limitation has been addressed by creating the mimetic RNActive vaccine, wherein protamine-directed RNA works as an immune stimulator rather than an expression vector [88].

#### Cationic lipid-polymer and polymeric carrier-based delivery

These are effective mixtures for transfecting mRNA, which are composed of cationic lipids or polymers like lipofectamine or TransIT-mRNA and have been shown to work effectively in numerous primary and cancer cell lines [37,112]. However, these are associated with partial *in vivo* efficacy and occasional toxicity. To overcome these limitations, there has been significant progress in designing safe and complex reagents for effective *in vivo* use [38,39,113,114]. Cationic lipids and polymers, with dendrimers, have been extensively utilized in mRNA processing in the past. The use of *in vivo* small interfering RNA (siRNA) delivery as a preferred method has greatly advanced the field of mRNA [115]. Among the most significant and commonly used methods for delivery of mRNA is using lipid nanoparticles (LNPs). These LNPs usually comprise three elements: (1) An ionizable cationic lipid that allows self-assembly into units of virus-like size, which facilitates endosomal escape of mRNA into the cytoplasm; (2) a lipid-polyethylene glycol (PEG) conjugate that prolongs the half-life duration of formulation; (3) cholesterol, a natural balancing component present in the phospholipids that maintains the structure of the lipid bilayer. Multiple studies have demonstrated that LNPs are an effective *in vivo* delivery method for siRNA.Systemic delivery of the mRNA-LNP complex which primarily targets the liver by attachment of apolipoprotein E followed by uptake through receptors in hepatocytes [116]. Intradermal, intramuscular, and subcutaneous vaccine routes result in extended expression of mRNA at the injection site. The mRNA mechanism wherein it enters the cytoplasm isn’t entirely known for the synthetic liposome nor naturally occurring exosomes. Additional investigation into this field is needed.

The duration for the *in vivo* protein formation from mRNA-LNP vaccines may be monitored for differing routes of administration. Intramuscular and intradermal vaccine mRNA-LNP delivery have demonstrated transient effects. For instance, in one of the experimental approaches, the half-life for mRNA coding for firefly luciferase remained barely prolonged post-intradermal injections than through intravenous therapy [117].

**Figure 4:**
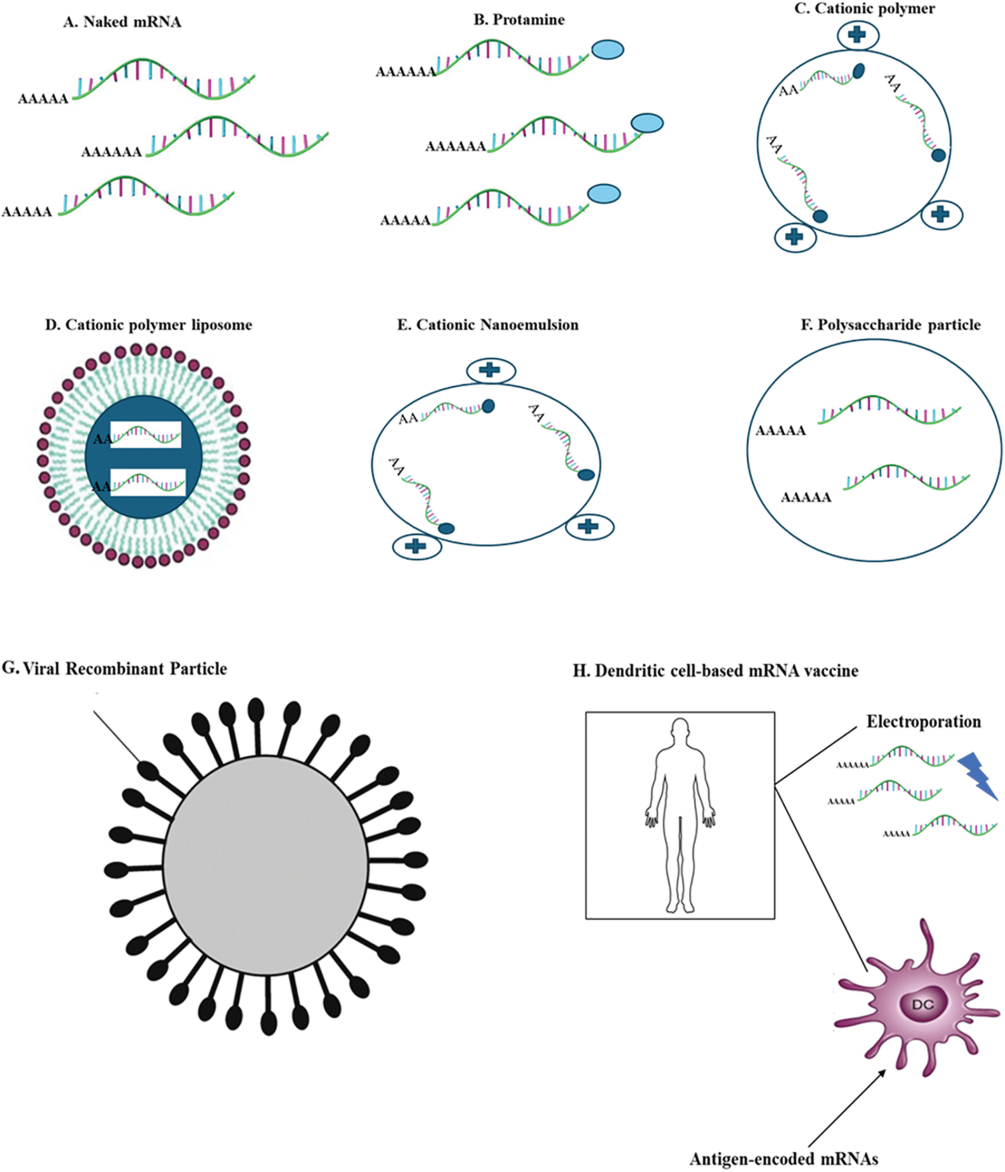
*Main delivery systems for carrying mRNA vaccines*; the figure depicts frequently used delivery techniques with carrier molecules having specific diameters for mRNA vaccines. (A) Naked mRNA (B) Protamine (C) Cationic polymer (D) Cationic polymer liposome (E) Cationic Nanoemulsion (F) Polysaccharide particle (G) Viral recombinant particle (H) Dendritic cell-based mRNA vaccine.

Studies investigating the kinetics of mRNA-LNP expression have shown promising results for generating immune responses. Prolonged antigen accessibility may be a factor that contributes to the effectiveness of vaccines using nucleoside-modified mRNA-LNP [77,79,117], resulting in increased antibody titers and responses from B cells located in the germinal center and T follicular helper cells [118,119]. T_FH_ cells constitute a critical subset of immune cells considered essential for vaccines to elicit robust and durable neutralizing antibody responses, particularly in the face of viruses that evade humoral immunity [118]. The dynamics of the GC reaction and segregation of T_FH_ cells are somewhat known, and enhancing knowledge in these regions could improve future vaccine designs (Fig. 4).

### mRNA-based vaccines for elimination and management of infectious diseases

To combat, as well as to avoid any epidemics, improvement of prophylactic or therapeutic vaccines countering virulent micro-organisms is highly desirable. Conventional vaccine development methods have been unsuccessful because they do not protect against complex viruses causing chronic or recurrent infections. Examples of viruses that vaccine development has not been successful include HIV-1, RSV, respiratory syncytial, and herpes simplex virus. Commercialization of vaccines and gaining regulatory approval is difficult as proven by the reaction to emergency outbreaks such as the Ebola-Zika virus in the year 2014–2016. Therefore the immediate rollout of an effective vaccine with regulatory approval is important [24].

Preclinical studies reveal that mRNA vaccines are potentially good vaccine candidates. These are tolerable in animals, quick to produce for evolving diseases, and easy to scale up using Good Manufacturing Practice (GMP) facilities.

In comparison to protein vaccines, various templates (such as single/double-stranded) for mRNA-based vaccines, stimulate potent CD8+ T cell reaction, due to the effectual presentation of endogenous antigens on MHC class I molecules and provide an effective CD4+ T cell response [70,86,93]. Moreover, mRNA vaccines have displayed the capacity to elicit more effective neutralizing antibody responses in animals by single or double-dosage immunizations [43,77,79]. mRNA vaccines have provided considerable optimism because they have generated an immune response against bacterial and viral infectious agents in mouse models [83,85,93,120].

As mentioned earlier, RNA vaccines against infectious diseases can be classified into two main types: (1) Self-amplifying or replicon RNA vaccines (SAM) and (2) non-replicating mRNA vaccines (NRM). Non-replicating mRNA vaccines have various administration methods including loading of DCs *ex vivo* and injecting into various anatomical sites *in vivo*.

### mRNA cancer vaccines

Currently, evaluations are being carried out for several mRNA cancer vaccines. Cancer vaccines along with other immunotherapies have shown encouraging therapeutic value to manage malignancies. These vaccines focus on tumor-associated antigens that occur in specially differentiated malignant cells, for instance: growth-associated factors or antigens that happen to be exclusive to cancerous cells arising from genetic mutations [121]. The neoantigen/epitopes are implemented as targets for mRNA vaccines focusing on humans [122]. Most of the vaccines stimulate cell-mediated responses, for instance, vaccines derived from CTLs, which decrease tumor burden [123]. The initial idea of RNA cancer vaccines being viable was observed more than two decades ago [124,125]. The practicality of using mRNA vaccines to prevent cancer has been shown through various pre-clinical and clinical studies (Refer to Table 2).

**Table 2:** mRNA cancer vaccine in clinical trial

Institution	Vaccine type	Objectives	Test numbers	Category
Argos therapeutics	Dendritic Cells electroporated along autologous tumor mRNA together with or minus CD40L mRNA (*i.d*.)	Renal cell carcinoma	• NCT01482949 (II)	Concluded [42]
			• NCT00678119 (II)	• Concluded; Results pending
			• NCT00272649 [126]	• Concluded; Results pending.
			• NCT01582672 [126]	
			• NCT00087984 [126]	
		Pancreatic carcinoma	NCT00664482 (NA)	Concluded; Results pending.
Antwerp university hospital	Dendritic Cells electroporated along tumor associated antigens mRNA (*i.d*. or NA)	Acute myeloid leukemia	• NCT00834002 (I)	• Concluded [127,128]
			• NCT01686334 (II)	• Recruiting
		Acute/Chronic myeloid leukemia, multiple myeloma	NCT00965224 (II)	Unknown
		Solid multiple tumors	NCT01291420 [126]	Unknown
		Mesothelioma	NCT02649829 [126]	Recruiting
		Glioblastoma	NCT02649582 [126]	Recruiting
Asterias biotherapeutics	Dendritic Cells laden along with tumor associated antigens mRNA	Acute myeloid leukemia	NCT00510133 (II)	Completed [129]
BioNTech RNA pharmaceuticals GmbH	Bare tumor associated antigen/neo-Ag mRNA (internodal)	Melanoma	NCT01684241 (I)	• Completed; Results pending
			NCT02035956 (I)	• Ongoing
	Liposome-combined *(i.v*.) tumor associated antigen mRNA	Melanoma	NCT02410733 (I)	Recruiting [62]
	Liposome-articulated tumor associated antigen/ neo-Ag mRNA (*i.v*.)	Breast carcinoma	NCT02316457 (I)	Recruiting
CureVac AG	RNActive tumor associated antigen mRNA (*i.d*.)	Non-small-cell lung cancer	• NCT00923312 [126]	• Completed
			NCT01915524 (I)	• Terminated [90]
		Prostate carcinoma	• NCT02140138 (II)	• Terminated
			• NCT00831467 [126]	• Concluded [92]
			• NCT01817738 [126]	• Terminated [130]
Duke University	Dendritic Cells laden with Cytomegalo virus antigen mRNA (*i.d*.)	Glioblastoma, malignant glioma	• NCT00626483 (I)	• Ongoing [131]
			• NCT00639639 (I)	• Ongoing [132,133]
			• NCT02529072 (I)	• Recruiting
			• NCT02366728 (II)	• Recruiting
	Dendritic Cells laden along autologous tumor mRNA (*i.d*.)	Glioblastoma	NCT00890032 (I)	Completed; Results pending
	Dendritic Cells developed, laden along tumor associated mRNA (*i.nod*.)	Melanoma	NCT01216436 (I)	Terminated
Erasmus medical center	Dendritic Cells laden along viral Ag mRNA combining TriMix (*i.nod*.)	Human immunodeficiency Virus-1	NCT02888756 (II)	Recruiting
Fundació Clínic per la Recerca Biomèdica	Viral antigen mRNA including TriMix	Human immunodeficiency Virus-1	NCT02413645 (I)	Underway
Guangdong 999 brain hospital	Dendritic Cells laden along tumor associated antigen mRNA	Glioblastoma	• NCT02808364 [126]	• Recruiting
			• NCT02709616 [126]	• Recruiting
		Brain metastases	NCT02808416 [126]	Recruiting
Herlev hospital	Dendritic Cells packed together through tumor associated antigen RNA (*i.d*.)	Breast carcinoma, melanoma	NCT00978913 (I)	Completed [134]
		Prostate cancer	NCT01446731 (II)	Completed [126]
Life research technologies GmbH	Dendritic Cells, developed, packed together tumor associated antigen mRNA	Ovarian carcinoma	NCT01456065 (I)	Unknown
Ludwig-Maximilian-University of Munich	Dendritic Cell packaged along cytomegalovirus antigen with tumor associated antigen mRNA (*i.d*.)	Acute myeloid leukemia	NCT01734304 [126]	Recruiting
MD anderson cancer center	Dendritic Cell packaged along lysate of AML along mRNA	Acute myeloid leukemia	NCT00514189 (I)	Terminated
Memorial sloan kettering cancer center	Dendritic Cells [135] electroporated along tumor associated antigen mRNA *(i.d*.)	Melanoma	NCT01456104 (I)	Ongoing
Massachusetts general hospital	Dendritic Cells packaged along viral antigen mRNA (*i.d*.)	Human immunodeficiency Virus-1	NCT00833781 (II)	Completed
McGill university health centre	Dendritic Cell Electroporated along homologous viral antigen as well as CD40L mRNAs (*i.d*.)	Human immunodeficiency Virus-1	NCT00381212 [126]	Completed
Moderna therapeutics	Nucleoside-altered viral antigen mRNA (*i.m*.)	Zika virus	NCT03014089 [126]	Recruiting
		Influenza virus	NCT03076385 (I)	Ongoing
Oslo university hospital	Dendritic Cells packed with homologous tumor/tumor associated antigen mRNA (*i.d*.)	Melanoma	• NCT00961844 [126]	• Terminated
			• NCT01278940 [126]	• Completed [136]
		Prostate carcinoma	• NCT01197625 [126]	• Recruiting
			• NCT01278914 [126]	• Completed; Results pending
		Glioblastoma	NCT00846456 [126]	Completed [137]
		Ovarian carcinoma	NCT01334047 [126]	Terminated
Universitair ziekenhuis Brussel	Dendritic Cells electroporated along tumor associated antigens with TriMix mRNA (*i.d*. as well as *i.v*.)	Melanoma	• NCT01066390 (I)	• Completed [138]
			• NCT01302496 (II)	• Completed [139]
			• NCT01676779 (II)	• Completed; Results pending
University hospital Erlangen	Dendritic Cells, grown, packaged along homologous tumor RNA (*i.v*.)	Melanoma	NCT01983748 [126]	Recruiting
University hospital Tübingen	Homologous tumor mRNA allied along GM-CSF protein (*i.d*. as well as *s.c*.)	Melanoma	NCT00204516 [126]	Completed [140]
	Protamine-combined tumor associated Antigen mRNA along GM-CSF protein (*i.d*. with *s.c*.)	Melanoma	NCT00204607 [126]	Completed [139]
University of Campinas, Brazil	Dendritic Cells packed along tumor associated antigens mRNA (NA)	Acute myeloid leukemia, myelodysplastic disorders	NCT03083054 [126]	Recruiting
University of Florida	RNActive* tumor associated antigen mRNA (*i.d*.)	Prostate carcinoma	NCT00906243 [126]	Terminated
	Dendritic Cells packed together to Cytomegalovirus Antigen allied to GM-CSF protein (*i.d*.)	Glioblastoma, malignant glioma	NCT02465268 (II)	Recruiting
Radboud University	Dendritic Cells electroporated together with tumor associated antigen mRNA (*i.d*. as well as *i.v*. either *i.nod*)	Colorectal carcinoma	NCT00228189 [126]	Completed [141]
		Melanoma	• NCT00929019 [126]	• Terminated
			• NCT00243529 [126]	• Completed [126,142]
			• NCT00940004 [126]	• Completed [142,143]
			• NCT01530698 [126]	• Completed [142–144]
			• NCT02285413 (II)	• Completed; Results pending

Note: *i.n,: internodal, i.m.: intermuscular, i.v; intravenous, i.d.: interdorsal, s.c.: subcutaneous*.

### DC mRNA cancer vaccines

Dendritic cells (DCs) elicit immune reactions specific to antigens, making them suitable for cancer immunotherapy. According to Boczkowski et al.’s 1996 study [125], DCs electropermeabilized with mRNA produce a robust immune response compared to tumor antigens. The research demonstrated that mice injected with DCs electroporated with ovalbumin (OVA)-coding mRNA displayed immune responses, which were also evident in other melanoma mouse models. Various immune regulatory proteins present in mRNA-coded adjuvants could augment the efficacy of DC-based cancer vaccines. Research has highlighted DCs electroporated with mRNAs coding for CD83, 4-IBBL, tumor necrosis factor receptor superfamily member 4 co-stimulatory molecules [145–148]. DC activities can be regulated via mRNA-translated cytokines, for instance, IL-12 or trafficking-linked molecules [149–151]. A mixture of mRNA-coded adjuvants (TL4, CD40L, CD70) with antigen-coding mRNAs also known as TriMix [152] can be electroporated. TriMiX showed effectiveness in several research studies by introducing DC along with modifying the CD4+ T cell subtype via regulatory T cell to helper T 1 cells [152–156]. Particularly, the inoculation of patients in later-stage cancer treatment utilizing DCs laden with mRNA coding for antigens associated with melanoma combined with TriMix adjuvant decreased tumor size by 27% [138]. Many clinical trials focusing on several cancers have utilized DC immunization: acute myeloid leukemia, brain cancer, melanoma, metastatic prostate & lung cancer [132,133]. As per clinical trials for advanced stage III or IV melanoma patients were given ipilimumab, a monoclonal antibody in contrast to CTL antigen 4 (CTL4) with DCs loaded with mRNA coding melanoma-related antigens with TriMix showed a reduction amongst recurring tumors [157].

### Administering mRNA cancer vaccines directly through injection

The method of administration along with the dispensing technique for mRNA-based vaccines can significantly impact results. There have been studies investigating administration routes employing common delivery methods; (transdermal, hypodermal, subdermal, or nasal) along with a few alternative methods of vaccinations (nodal, IV, liver, or intratumorally).

Although administration via the nodal route of bare mRNA is unusual, it is an effective way for delivery of vaccine. Directly injecting mRNA into peripheral lymphatic tissue extends the benefit towards direct delivery of the antigen near APCs adjacent to T cell activation preventing DC migration. Various studies have shown DCs can selectively take up naked mRNA obtaining effective preventive or therapeutic anti-cancer T cell reactions [99,103]. Initial research via intrasplenic delivery showed this finding [76]. A combination of DC-stimulating proteins FMS-associated tyrosine kinase 3 ligand (FLT3L) demonstrated additional improvement within immune response towards intranodal vaccination mRNA route [33,158]. Integration of the TriMix adjuvant within the intranodal dose of injections in mice along with mRNAs coding for cancer-specific antigens leads to tumor control in several tumor models with effective antigen-specific CTL responses [153]. A recent case study with vaccination via an intranodal route for mRNA coding for the E7 protein of human papillomavirus showed inhibition of E7-articulating tumor models with an increase in CD8+ T cells [104].

Preclinical studies have shown success with intranodal injection of exposed mRNA coding for tumor-associated antigens in patients having advanced melanoma and are presently in clinical trials. In a study of metastatic melanoma treatment with intranodal DCs electroporated by mRNAs coding for antigens which are melanoma-associated tyrosine or gp100 with TriMix stimulating inhibited antitumor reactions [144].

The intranasal route of vaccine administration is a needle-free, non-invasive approach for vaccine delivery which allows quick antigen absorption by DCs. Intranasal delivery of mRNA combined with Stemfect LNPs impeded tumor onset with enhanced continuity in prophylactic and therapeutic *in vivo* tumor prototypes utilizing OVA-expressing E.G7-OVA in T lymphocyte cell line [65]. Intratumoral mRNA immunization has been demonstrated to be beneficial because of the specific initiation of tumor-resident T cells [24].

Often, the administration of vaccines fails to induce an immune response. Instead, mRNA molecules encoding tumor-associated antigens are directed to stimulate tumor-specific immunity on-site by utilizing immune-stimulating particles. Initial studies indicated exposed mRNA protamine-stable mRNA coding for a non-tumor associated gene (GLB1) reduced tumor growth and gave protection in a glioblastoma mice study by considering the intrinsic immunogenic properties of mRNA [159]. Another interesting study revealed the intratumoral delivery of mRNA coding for a modified cytokine confirmed the presence of interferon B cells tagged to a transforming growth factor.

The presence of interferon B cells enhanced the catalytic activity of CD8+ T cells, thereby suppressing tumor advancement in lymphoma overexpressing OVA [160]. Moreover, it has been observed that TriMix RNA intratumoral administration not coding for tumor-associated antigen leads to initiation of CD8+ DCs with specific tumor T cells inhibiting tumor progression *in vivo* [161].

Preparing mRNAs attached to carrier molecules is important, as successful uptake of mRNA vaccines is based on preventing the accumulation of specific serum proteins and extracellular breakdown of mRNA. As mentioned above there are various delivery platforms that have been established for enabling mRNA absorption, improving protein translation, along with protecting mRNA from RNAses [38,39,113,114]. Moreover, the biodistribution of mRNA vaccines post-systemic administration is another major issue. Specific cationic LNP-based complexing mediators target intravenously primarily towards the liver [117], and might not be best for initiation of DC. An efficient approach for DC aiming mRNA vaccines post-systemic distribution lately has been described [62]. The mRNA-lipoplex (mRNA-liposome compound) transporting platform, used cationic lipids with neutral helper lipids attached to mRNA. It has been identified that the mRNA lipid ratio promoting the net charge of the particle has a great effect on the biodistribution of the vaccine.

A diverse range of antigen-presenting cells can be found within the skin membrane, making it a common site for immunogen administration through vaccination [135]. Therefore, the intradermal route is widely utilized for delivering m RNA cancer vaccines. A case report established that intradermal processing of tumor RNA led to prolonged tumor growth in a fibrosarcoma mouse model [102]. In various cancer mouse models, mRNA intradermal injections coding for tumor antigens within protamine based RNActive programs showed efficacy in both preventive and therapeutic contexts. Another study involving patients with melanoma showed that mRNA encoding survivin and other melanoma-associated tumor antigens led to the creation of a greater number of antigen-specific T-cells [89]. In patients having castrate-resistant prostate cancer, an RNActive vaccine targeting several prostate cancer-associated proteins produced an antigen-specific T-cell response [92]. Lipid-based carriers have also proven effective for intradermal mRNA vaccinations. Release of OVA-coding mRNA in DOTAP/DOPE liposomes induced an antigen-specific cytotoxic T lymphocyte [9] response leading towards reduced progression of OVA-expressing tumors in an *in vivo* model [9]. Additionally, the use of mRNA expressing granulocyte-macrophage colony-stimulating factor (GM-CSF) in combination with OVA enhanced cytolytic reactions. Subcutaneous administration of mRNA coding for melanoma-associated antigens using lipid nanoparticle (LNP) delivery hindered tumor growth *in vivo*, especially when co-administered with lipopolysaccharide within LNPs, thereby improving CTL and antitumor function [162]. Overall, mRNA-based cancer vaccines have demonstrated immunogenic responses in individuals, but further advancements in vaccine development hold the potential for greater clinical success.

### Specific cancer vaccine approach

In recent decades, a significantly improved comprehension of how cancer cells evade detection by the immune system has resulted in notable advancements in cancer immunotherapy. Both immune checkpoint inhibitors and adoptive cell therapies have shown the capability to induce tumor regression in patients with haematological and solid tumors [163]. These breakthroughs have highlighted the potential of tumor immunotherapy, and the development of cancer vaccines has entered a period of rapid expansion, aiming to replicate natural immunity. Therapeutic cancer vaccines mark a novel direction in the realm of upcoming immunotherapy, mainly due to their safety, specificity, and capacity to establish lasting immune memory. Globally, there is a considerable upswing in early-stage clinical research on cancer vaccines [164]. While previous studies have examined predefined antigen cancer vaccines focused on TAA, their effectiveness is constrained. Notably, there is substantial interest in personalized cancer vaccines that target neoantigens [165]. Yet, their broad implementation faces limitations due to hurdles like the complexity of identifying neoantigens, challenges in swift production, and the complexities of detecting clinically significant immune responses. Insights gleaned from less successful clinical trials have proven valuable, guiding necessary adjustments and modifications to improve their efficacy [166]. These efforts highlight the dynamic nature of research and the iterative process necessary to advance cancer vaccines toward realizing their complete therapeutic capabilities. The future enhancement of the clinical effectiveness of therapeutic cancer vaccines is focused on several critical areas, such as pinpointing immunogenic neoantigens, refining vectors and delivery mechanisms, and overcoming the immunosuppressive tumor microenvironment (TME) [163]. Advanced technologies including next-generation sequencing, augmented computing capabilities, and sophisticated algorithms have greatly facilitated the identification of highly immunogenic neoantigens. Continuous research is committed to enhancing vaccine technologies, which involves investigating various expression formats, enhancing co-stimulation components, and identifying appropriate prime-boost strategies. The careful selection of a delivery system is essential to enhance the immunogenicity of antigens and facilitate the entry of both the antigen and an activation signal into antigen-presenting cells (APCs) [167]. Another avenue of exploration involves strategically integrating immune checkpoint inhibitors and other treatment protocols. These combined approaches have the potential to counteract the immunosuppressive environment of the tumor microenvironment (TME) and alleviate the development of resistance to immunotherapy. However, further investigation and refinement are needed to determine the precise composition, sequence, and dosage of combination therapies [168].

Creating a cytotoxic T-cell response specific to tumors remains a crucial obstacle in cancer immunotherapy. The primary goal of these complex strategies is to choose a treatment plan that induces robust, long-lasting, and tumor-specific immunity in cancer patients, thereby extending their survival through cancer vaccines. The integration of innovative methods, careful selection of candidates, and improved administration protocols have the potential to transform cancer treatment, ushering in a new era of therapeutic cancer vaccines. With ongoing progress, we anticipate the successful development of numerous therapeutic cancer vaccines [169].

### The outlook for mRNA cancer vaccines

mRNA-driven cancer vaccines are emerging as a promising solution, offering versatility, potency, scalability, precision, cost-effectiveness, and freedom from cold chain requirements. They address various challenges associated with DNA vaccines and can be produced on a large scale for clinical use. The growing number of clinical trials exploring cancer treatments highlights heightened industry interest in bringing mRNA-based cancer vaccines to market [24]. However, achieving this goal requires a sustainable and cost-effective manufacturing process that addresses three primary concerns: instability, innate immunogenicity, and inefficient *in vivo* mRNA delivery. The mechanism of an mRNA vaccine differs from that of a traditional vaccine. Vaccines work by stimulating the body’s immune system to build defenses against specific diseases. Traditional vaccine approaches involve introducing a weakened or inactivated version of the virus or bacteria itself. This exposure allows the immune system to recognize and mount a response against the pathogen if encountered in the future [170].

In contrast, mRNA vaccines take a different approach to presenting antigens to the immune system. Rather than containing the entire pathogen or selected components, mRNA vaccines provide instructions that allow the body’s cells to manufacture the specific viral protein antigen. This antigen production within our cells then triggers the desired immune response and antibody generation. Instead of injecting the antigen directly, the mRNA directs our cells to produce it themselves. This novel mechanism sets mRNA vaccines apart from longstanding vaccine strategies using attenuated or inactivated pathogens or subunit protein antigens [171].

mRNA cancer vaccines represent an exciting new frontier in cancer treatment that offers both opportunities and challenges. Their ability to be personalized and precisely target tumor-specific antigens makes them a promising approach for precision oncology. However, more clinical trials are needed to fully establish their safety and efficacy profiles. Further preclinical research is also warranted to investigate potential synergies between mRNA cancer vaccines and other anticancer therapies. Overcoming hurdles such as tumoral heterogeneity, optimal delivery routes, and developing methods to evaluate therapeutic efficacy will be crucial steps toward translating this technology into meaningful clinical outcomes. With sustained research efforts and resources dedicated to this field, mRNA cancer vaccines hold immense potential to transform cancer care and provide new hope for patients battling this devastating disease [157].

## Discussion

mRNA technology represents a groundbreaking advancement in the field of biotechnology with far-reaching implications for medicine, science, and beyond. Its rapid development, particularly exemplified by the successful deployment of mRNA vaccines against the COVID-19 pandemic, has demonstrated its immense potential to revolutionize the way we prevent, treat, and potentially cure diseases. The versatility, safety, and efficacy demonstrated by mRNA-based therapies herald a new era in healthcare, where precision medicine tailored to individual genetic profiles becomes increasingly feasible. Advancements in mRNA structure modification techniques, including nucleotide modifications and codon optimizations, along with novel purification methods, have the potential to enhance the internalization of mRNA by antigen-presenting cells (APCs). Additionally, the innate immunogenicity of mRNA can serve as an adjuvant-like effect to enhance the immune response. However, the paradoxical nature of intrinsic immunity mediated by interferon-related pathways may lead to mRNA degradation, resulting in reduced antigen expression [58,61]. Innovative formulation approaches such as LNPs, CARTs, and CPPs hold significant promise in enhancing delivery efficacy. Therefore, optimizing the mRNA sequence, the purity of mRNA products, the delivery system, and administration routes are crucial for activating an appropriate immune response without inducing toxic overreactions.

mRNA-based cancer vaccines stimulate anti-tumor immunity, triggering antibody responses, B cell-mediated humoral reactions, and CD4+/CD8+ T cell responses [66,172]. Currently, three primary types of RNAs are utilized as cancer vaccines: non-replicating unmodified self-amplifying mRNAs (SAMs), modified SAMs, and virus-derived SAMs [173]. Among these, SAMs are extensively researched in both cancer and infectious diseases due to their sustained efficacy and economical dosage. SAMs originate from positive single-stranded mRNA viruses [174]. They have the capacity to self-replicate for as long as 2 months, leading to a heightened and enduring immune response. Additionally, the SAMs platform generates a significant quantity of antigens over an extended period, necessitating lower vaccine doses [24]. Despite garnering considerable interest and serving as a promising alternative to mRNA-based vaccines, SAMs’ clinical use in cancer treatment is currently restricted to preliminary evaluations of viral replication particles [175].

The emergence of neoantigens has led to the rise in popularity of personalized vaccines, often paired with checkpoint blockade modulators or cytokine cocktails. This combination aims to enhance the host’s anti-tumor immunity and improve the chances of eliminating tumor cells [176,177]. Personalized neoantigen-based cancer vaccines represent tumor-specific treatments. In contrast to tumor-associated antigens, neoantigen-specific T-cells are more likely to persist despite immune self-tolerance progression. This persistence enhances robust T-cell responses and increases the breadth and diversity of the immune response.

Furthermore, these vaccines target a variety of variant mutations to mitigate off-target effects, thereby improving safety [178]. Moderna, for instance, developed an mRNA-based personalized cancer vaccine comprising the patient’s unique tumor neoantigens. This vaccine targets approximately 20 neoepitopes, representing mutations specific to the patient’s cancer cells. Following administration to the patient, the vaccine prompts the patient’s cells to express these specific neoepitopes, aiding the immune system in distinguishing cancer cells from normal cells. Consequently, the patient’s immune system becomes more adept at recognizing and eliminating cancer cells. Moreover, combining mRNA-based cancer vaccines with other therapies can bolster anti-tumor effects. In 2016, Moderna and Merck collaborated with the intention of combining mRNA-4157, which is linked to checkpoint inhibitor therapies, with the anti-PD-1 therapy KEYTRUDA. Presently, a phase I study has been undertaken to assess the safety, tolerability, and immunogenicity of mRNA-4157 alone in individuals with resected solid tumors, as well as the combined approach with KEYNOTE-603 in individuals with unresectable solid tumors [179]. Additionally, BioNTech is conducting two phase II studies to evaluate the efficacy, tolerability, and safety of mRNA-based cancer vaccines in the treatment of patients with anti-PD-1-refractory/relapsed unresectable stage III or IV melanoma and colorectal cancer patients who have undergone surgery and chemotherapy, respectively [180]. The combination of BNT111 with Libtayo is anticipated to trigger a robust and targeted immune response against cancer. On the other hand, BNT122 is viewed as a personalized treatment for colorectal cancer patients, customized to align with the unique tumor characteristics of each individual, potentially leading to reduced costs. Looking ahead, the future benefits of mRNA technology are vast and multifaceted. Firstly, the adaptability of mRNA platforms enables swift responses to emerging infectious diseases, potentially mitigating future pandemics with targeted vaccine development. Additionally, mRNA vaccines offer promising avenues for combating a wide range of diseases, including cancer, infectious diseases, autoimmune disorders, and genetic conditions, offering hope to millions of patients worldwide.

Furthermore, mRNA technology holds the promise of personalized medicine, where therapies can be tailored to an individual’s unique genetic makeup, maximizing efficacy while minimizing adverse effects. This could revolutionize treatment paradigms across numerous medical fields, leading to more effective and personalized healthcare interventions.

Beyond healthcare, mRNA technology has the potential to drive innovation in other sectors, such as agriculture and environmental remediation, by enabling the development of novel biotechnological solutions. However, despite these promising applications, there are several limitations associated with the article that need to be addressed for a comprehensive understanding of this technology; firstly, being limited long-term data; as m RNA technology is relatively new, long-term efficacy and safety data re limited. Secondly data transparency and accessibility: Not all clinical trial data and real-world evidence are fully transparent or accessible. Proprietary information and unpublished data can limit the scope of the review, potentially omitting critical insights necessary for a comprehensive analysis. Thirdly, heterogeneity of studies; the studies included in the review may vary significantly in their design, methodology, and population demographics. This heterogeneity makes it difficult to draw consistent and generalized conclusions, as results may not be directly comparable across different studies. While mRNA vaccines represent a significant advancement in vaccine technology, the review of their development and application is constrained by various limitations. Addressing these challenges requires ongoing research, transparency, and collaboration within the scientific community to ensure comprehensive, accurate, and up-to-date evaluations.

## Conclusion

In summary, the continued advancement and application of mRNA technology offer a bright future characterized by improved health outcomes, personalized treatments, and innovative solutions to pressing global challenges. As research and development in this field progress, the full extent of mRNA’s transformative potential is yet to be realized, promising a future defined by unprecedented possibilities for human health and well-being.

## Data Availability

Data sharing is not applicable to this article as no datasets were generated or analyzed during the current study.

## Abbreviation

mRNA: Messenger RNA
APCs: Antigen-Presenting Cells
LNPs: Lipid Nanoparticles
FDA: Food and Drug Administration
CAR: Chimeric Antigen Receptor
TAA: Tumor-Associated Antigen
TSA: Tumor-Specific Antigen
HPV: Human Papillomavirus
HBV: Hepatitis B Virus
HLA: Human Leukocyte Antigen
mRNA: Messenger RNA
IVT: *In Vitro* Transcription
SAM: Self-Amplifying mRNA
COVID-19: Coronavirus Disease 2019
MHC: Major Histocompatibility Complex
ORF: Open Reading Frame
UTR: Untranslated Region
dsRNA: Double Stranded DNA
PAMPs: Pathogen-Associated Molecular Patterns
PRRs: Pattern Recognition Receptors
TLR: Toll-Like Receptor
MyD88: Myeloid Differentiation Marker 88
IFN: Interferon
RIG-I: Retinoic Acid-Inducible Gene-I
OAS: Oligoadenylate Synthetase
PKR: RNA-Dependent Protein Kinase
ssRNA: Single Stranded RNA
DC: Dendritic Cells
eIF-2: Eukaryotic Initiation Factor-2
IFNAR: Interferon-α/β Receptor
ARCA: Anti-Reverse Cap Analogs
IFIT: Interferon-Induced Proteins with Tetratricopeptide Repeats
PABP: Poly(A) binding protein
HPLC: High-Pressure Liquid Chromatography
FPLC: Fast Protein Liquid Chromatography
STING: Stimulator of Interferon Genes
CD40L: CD40 Ligand
VRP: Viral Replication Particles
PEI: Polyethylenimine
RSV: Respiratory Syncytial Virus
GM-CSF: Granulocyte-Macrophage Colony-Stimulating Factor
RVG: Rabies Virus Glycoprotein G
LNP: Lipid Nanoparticle
DOTAP: 1,2-dioleoyl-3-trimethylammonium-propane
DOPE: 1,2-dioleoyl-sn-glycero-3-phosphoethanolamine
DSPC: 1,2-distearoyl-sn-glycero-3-phosphocholine
PEG: Polyethylene Glycol
EPO: Erythropoietin
DOE: Design of Experiment
PAMAM: Polyamidoamine
OVA: Ovalbumin
PBAE: Poly(beta-amino) Esters
APE: Amino Polyesters
pABOL: Poly(CBA-co-4-amino-1-butanol)
CART: Charge-Altering Releasable Transports
CPP: Cell-Penetrating Peptides
CFTR: Cystic-Fibrosis Transmembrane Regulator
CNE: Cationic Emulsions
DOTMA: 1,2-di-O-octadecenyl-3-trimethylammonium propane
LCP: Lipid/Calcium/Phosphate
LPR: Lipid-Polymer-RNA Lipopolyplexes
NSCLC: Non-Small Cell Lung Cancers
caTLR4: Constitutively Active TLR4
LAMP: Lysosomal-Associated Membrane Protein
CMV: Cytomegalovirus
SLP: Synthetic Long Peptide
